# The Role of *Pseudomonas aeruginosa* Virulence Factors in Cytoskeletal Dysregulation and Lung Barrier Dysfunction

**DOI:** 10.3390/toxins13110776

**Published:** 2021-11-02

**Authors:** Brant M. Wagener, Ruihan Hu, Songwei Wu, Jean-Francois Pittet, Qiang Ding, Pulin Che

**Affiliations:** 1 Department of Anesthesiology and Perioperative Medicine, University of Alabama at Birmingham, Birmingham, AL 35294, USA; bwagener@uabmc.edu (B.M.W.); huruihancool@163.com (R.H.); swu@uabmc.edu (S.W.); jpittet@uabmc.edu (J.-F.P.); qiangding@uabmc.edu (Q.D.); 2 Division of Molecular and Translational Biomedicine, University of Alabama at Birmingham, Birmingham, AL 35294, USA; 3 Division of Critical Care Medicine, University of Alabama at Birmingham, Birmingham, AL 35294, USA; 4 Center for Free Radical Biology, University of Alabama at Birmingham, Birmingham, AL 35294, USA; 5 Department of Internal Medicine, Guiqian International General Hospital, Guiyang 550024, China

**Keywords:** *Pseudomonas aeruginosa*, virulence factors, actin cytoskeleton, lung barrier integrity

## Abstract

*Pseudomonas (P.) aeruginosa* is an opportunistic pathogen that causes serious infections and hospital-acquired pneumonia in immunocompromised patients. *P. aeruginosa* accounts for up to 20% of all cases of hospital-acquired pneumonia, with an attributable mortality rate of ~30–40%. The poor clinical outcome of *P. aeruginosa*-induced pneumonia is ascribed to its ability to disrupt lung barrier integrity, leading to the development of lung edema and bacteremia. Airway epithelial and endothelial cells are important architecture blocks that protect the lung from invading pathogens. *P. aeruginosa* produces a number of virulence factors that can modulate barrier function, directly or indirectly, through exploiting cytoskeleton networks and intercellular junctional complexes in eukaryotic cells. This review summarizes the current knowledge on *P. aeruginosa* virulence factors, their effects on the regulation of the cytoskeletal network and associated components, and molecular mechanisms regulating barrier function in airway epithelial and endothelial cells. A better understanding of these processes will help to lay the foundation for new therapeutic approaches against *P. aeruginosa*-induced pneumonia.

## 1. Introduction

**Figure 1:**
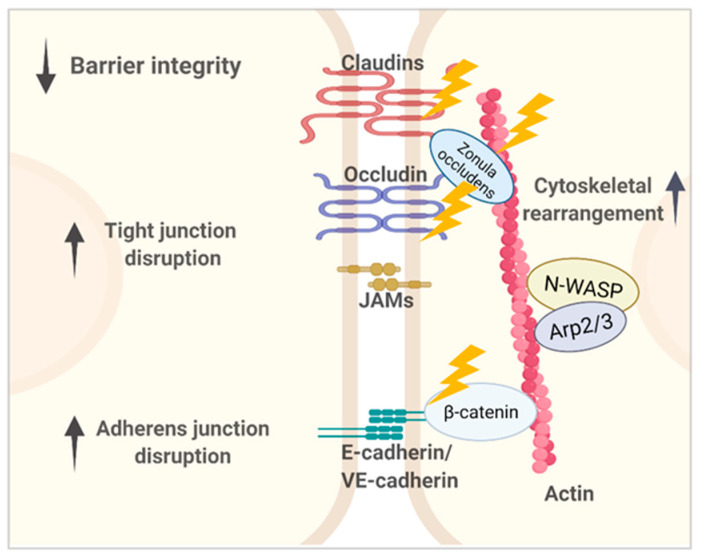
Representation of lung microvascular barriers composed of the major intercellular junctional structures, including tight junctions (TJs) and adherens junctions (AJs). Epithelial AJs contain epithelial cadherin (E-cadherin), and endothelial AJs contain vascular endothelial cadherin (VE-cadherin). The main structural components and their cytoplasmic partners involved in *P. aeruginosa*-induced lung barrier dysregulation are shown. *P. aeruginosa* virulence factors impair paracellular permeability through disrupting junctional components’ expression, redistribution, and interaction with adaptors that together are key to maintain proper junctional structure and function. Compromised epithelial and endothelial barriers eventually contribute to *P. aeruginosa* dissemination from the lung into the bloodstream.

*Pseudomonas (P.) aeruginosa* is an opportunistic pathogen that causes serious infections and hospital-acquired pneumonia (HAP) in at-risk patients, such as those with compromised immune system, who are post-surgical and admitted to intensive care units (ICUs) [1,2,3,4,5]. *P. aeruginosa* accounts for up to 20% of all cases of HAP, with an attributable mortality rate of ~30–40% [6,7,8,9]. The devastating outcome is associated with lung barrier destruction, which permits lung edema formation and *P. aeruginosa* bacteremia, a poor prognostic sign [10,11]. Airway epithelium and endothelium constitute a continuous barrier that protect the lung against respiratory pathogens. *P. aeruginosa* disrupts this protective layer by mechanisms targeting the components involved in regulating actin cytoskeletal networks, including proteins associated with the intercellular junctional complex. Lung alveoli, composed of a single layer epithelial cells, are relatively vulnerable in response to *P. aeruginosa* infection compared to other epithelium, such as gut and skin epithelium. Once alveolar epithelium is breached, *P. aeruginosa* and its secreted effectors can get into lung interstitium and interact with endothelial cells. *P. aeruginosa* may then access the blood stream by crossing endothelium and can cause bacteremia and even sepsis. Figure 1 shows the representation of epithelial and endothelial barriers composing the alveolar–capillary barrier. Evidence of *P. aeruginosa*-mediated alteration of intercellular junctional components suggests that *P. aeruginosa* transmigration through cell–cell junctions may be the main route for *P. aeruginosa* to enter the host system [12,13]. However, the mechanisms by which *P. aeruginosa* directly or indirectly disrupts junction integrity are still not fully understood.

The actin cytoskeletal network is involved in multiple physiological functions and plays a vital role in lung barrier integrity. One important function of the actin cytoskeleton is the maintenance of intercellular junctional structures through coordinating the interactions between components contained within the junctional complex. *P. aeruginosa* possesses a full arsenal of virulence factors. During *P. aeruginosa* infection, these virulence factors interrupt lung barrier functions through modulation of cytoskeletal regulators and generating contractile forces. These contractile forces compete with cell–cell tethering forces between adjacent cells and generate tension delivered to intercellular contacts, leading to the formation of gaps between contacting cells and increasing paracellular permeability as a consequence. Adherens junctions (AJs) and tight junctions (TJs), the primary intercellular complexes that control the lung barrier functions, are linked to and coordinate with the actin cytoskeleton. By exploiting the cytoskeletal network, *P. aeruginosa* not only disturbs junctional stability but also disrupts signal transduction that is fundamental to cellular functions, such as those performed by Rho GTPases. Moreover, the consequences of dysregulated cytoskeleton regulators go beyond the cytoskeleton alterations and lung barrier dysfunction, as some cytoskeleton regulators are involved in modulation of immune responses. For example, aberrant Rho GTPases contribute to transcriptional regulation of important inflammatory mediators as well as cytokine expression [14,15]. Thus, the actin cytoskeleton and its associated components play important roles in fine-tuning cell–cell junction functions and signal transduction as well as providing multiple targets for *P. aeruginosa* virulence factors. 

This review summarizes the current knowledge relating *P. aeruginosa* virulence factors, their interactions and effects on the cytoskeleton network and associated component proteins, as well as molecular mechanisms regulating lung barrier function in airway epithelial and endothelial cells. A thorough understanding of these processes will provide a foundation for new therapeutic approaches for *P. aeruginosa*-induced pneumonia and the development of therapies for lung barrier modulation. 

## 2. *P. aeruginosa* Regulation of the Cytoskeletal Network in Lung Epithelial Cells

The respiratory epithelium serves as the dominant barrier against *P. aeruginosa* invasion [16,17,18]. Intact epithelium has strictly controlled paracellular permeability due to the presence of intercellular junctions, primarily tight junction (TJs), and adherens junctions (AJs) [19,20]. However, impaired TJs and AJs allow pathogens and large macromolecules to move through the space between adjacent cells and, thus, penetrate through the epithelium. The regulation of epithelial paracellular permeability depends on a set of specialized adhesive membrane proteins arranged to precisely coordinate with actin cytoskeletal dynamics. Thus, the cytoskeleton plays an important role in physically and functionally balancing this regulatory network, as any imbalance may result in perturbed intercellular junctional stability and permeability. 

Many *P. aeruginosa* virulence factors modulate lung epithelial permeability through manipulation of cytoskeletal dynamics and associated regulators that alter protein expression, distribution, degradation, or phosphorylation status [21,22,23,24,25]. The Rho family of small GTPases plays a fundamental role in the regulation of actin cytoskeleton reorganization [26,27]. *P. aeruginosa* promotes permeability in epithelial and endothelial cells through activation of the upstream GTPase Ras homolog gene family, member A (Rho A) [28,29]. ExoS and ExoT, type 3 secretion system (T3SS) exoenzymes that are directly injected into host cells by *P. aeruginosa*, interfere with actin reorganization through hijacking the host eukaryotic Rho GTPase pathway [29,30,31]. Type III exoenzymes also trigger substantial redistribution of occludin (OCLN), zonula occludens-1 (ZO-1), and another TJs protein ezrin in human airway cells [25]. Besides targeting cell–cell junctions, *P. aeruginosa* utilizes certain integrins, such as integrin α5β1 and αvβ5, for attachment and invasion to airway epithelial cells [32,33]. TGF-β1 is a critical mediator of *P. aeruginosa*-induced acute lung injury [34], as integrin-mediated TGF-β1 activation contributes to signal transductions involved in cytoskeletal rearrangement and tyrosine kinase activation. For example, integrin αvβ6-mediated activation of TGF-β1 is essential for *P. aeruginosa*-mediated lung barrier disruption and edema formation [35,36]. 

*P. aeruginosa* targets tight junction components. Tight junctions (TJs), the first line of junctional defense that gate the paracellular pathway, are composed of transmembrane proteins, including claudins (CLDN), OCLN, tricellulin, and the junctional adhesion molecule (JAM) proteins [37,38,39,40,41]. The intracellular domains of these proteins are linked to actin cytoskeleton through adaptor proteins. such as ZO-1, ZO-2, ZO-3, and cingulin. *P. aeruginosa* induces decreased expression of TJs proteins, including claudin-1 and OCLN, resulting in reduced transepithelial electrical resistance (TER) across cultured airway cells [42]. *P. aeruginosa* elastase downregulates expression of claudin-1, claudin-4, OCLN, and tricellulin [21,22,43]. In addition, *P. aeruginosa* has been reported to stimulate OCLN and E-cadherin redistribution and E-cadherin cleavage through toll-like receptor 2 (TLR-2)-dependent activation of calpain [44]. 

Furthermore, *P. aeruginosa* targets the adherens junction components. Actin cytoskeleton plays an important role in providing contractility for lateral adhesions between adjacent cells by forming parallel actin bundles at the AJs [45,46,47,48,49]. AJs are mainly composed of clustered E-cadherin transmembrane proteins, which are linked to the actin cytoskeleton through catenins and vinculin. *P. aeruginosa* virulence factors modulate AJs integrity through regulation of expression and phosphorylation status of E-cadherin and beta-catenin [24,43,50,51]. 

Additionally, *P. aeruginosa* targets the adaptor proteins that connect the actin cytoskeleton with the intercellular junctional complex. At least 40 different proteins are known to localize to TJs, and only 4 TJs proteins are known to directly bind to actin cytoskeleton, namely ZO-1, ZO-2, ZO-3, and cingulin. ZO proteins bind directly to actin filaments (F-actin) [52,53], linking actin cytoskeleton with TJs transmembrane proteins, such as CLDNs, OCLN, and JAM [54,55,56]. ZO-1 has been shown to link with regulators involved in myosin II activity and Rho GTPase signaling [57,58], which are both important mediators of cytoskeletal dynamics. Lipopolysaccharide (LPS)-induced F-actin rearrangement is essential for LPS signaling [59]. *P. aeruginosa* LPS increases F-actin formation in a ZO-1-dependent pathway. Overexpression of ZO-1 has been reported to increase F-actin polymerization driven by PDZ-1 domain-mediated binding to claudins [60,61]. 

*P. aeruginosa*-induced effects on epithelial barrier functions are, at least partly, regulated through cytoskeleton reorganization. such as Rho GTPases regulated actin polymerization [29,30]. Further studies are needed for a molecular-level understanding of how *P. aeruginosa-*induced actin cytoskeleton reorganization affects TJs and AJs assembly and its impact on paracellular permeability.

## 3. *P. aeruginosa* Targets Cytoskeletal Network in Lung Endothelial Cells

Endothelial cells are specialized cells that line the internal surface of blood vessels and are responsible for the maintenance of vascular permeability. Although serving as a barrier between blood and interstitial fluid, the lung endothelium is composed of a single layer of endothelial cells, making it vulnerable to attack by *P. aeruginosa* virulence factors. Following disruption of the epithelial barrier, *P. aeruginosa* virulence factors have access to the endothelium, where proteases and toxins released from *P. aeruginosa* further disrupt endothelial tight junctions [62]. As a consequence of dysregulated endothelial cell barriers, *P. aeruginosa* can migrate into the bloodstream and lead to bacteremia and cause a fatal outcomes [63]. However, compared to airway epithelium, a small number of studies have investigated the destructive effects of *P. aeruginosa* virulence factors on lung endothelium [28,50,62,64,65]. Endothelium presents similar yet distinct intercellular junctional components when compared to those of the epithelium. For example, instead of E-cadherin expressed by epithelial AJs, endothelial AJs present VE-cadherin, an endothelial-specific cadherin [66,67]. *P. aeruginosa* elastase cleaves VE-cadherin [21,62]. Moreover, ExoS and ExoT increase paracellular permeability across endothelial cell monolayers through integrin αvβ5 with activation of RhoA signaling [28,68,69]. In addition, compared to the junctional complex in epithelium, the endothelium presents intermingled TJs and AJs [70]. Interestingly, recent evidence suggests that actin assembly at TJs and AJs are regulated through distinctive mechanisms [71,72]. Lung endothelium and epithelium also share some similar mechanisms in the role of cytoskeleton dynamics in barrier function in response to *P. aeruginosa* infection. Neural Wiskott–Aldrich syndrome protein (NWASP) plays a critical role in cytoskeleton dynamics and regulates barrier integrity through Rho GTPase signaling and cytoskeletal reorganization in lung endothelial and epithelial cells in response to *P. aeruginosa* and transforming growth factor beta-1 [64,73]. It has recently been noted that barrier function is more strictly controlled with 10 times higher transendothelial electrical resistance and more developed intercellular junctions in lung microvascular endothelium in comparison to lung macrovascular endothelium [74,75,76,77]. Additional studies are needed to understand the molecular mechanisms by which *P. aeruginosa* virulence factors breach the lung microvascular endothelium by modulation of cytoskeletal structures and cytoskeletal regulatory proteins.

## 4. Cytoskeletal Regulation by *P. aeruginosa* Virulence Factors

### 4.1. Regulation of Lung Permeability by Virulence Factors Belonging to P. aeruginosa Type III Secretion System

**Figure 2:**
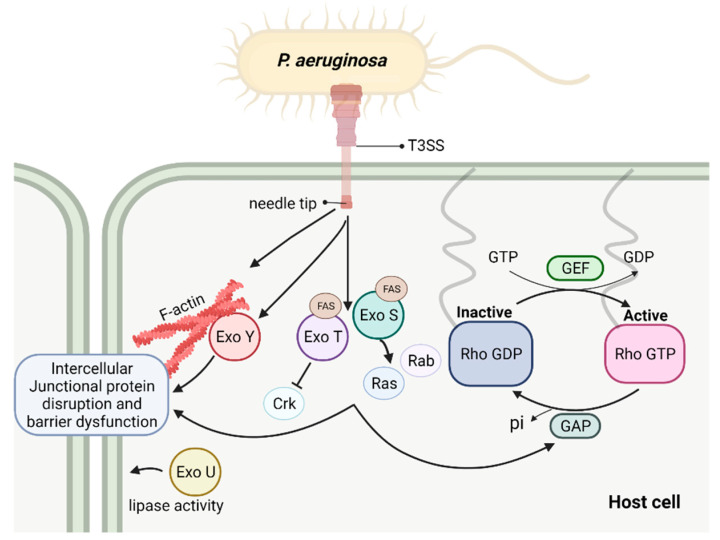
Schematic depicting T3SS exoenzymes and their interaction with host intracellular pathways contributing to barrier disruption. These events result in actin stress fiber formation, cytoskeleton rearrangement, and disruption of intercellular junctions, following with increased permeability.

Type III secretion system (T3SS) is the major contributor to *P. aeruginosa*-induced virulence [78,79,80,81]. Epithelial cells are especially sensitive to the effects of T3SS toxins [25,80,81,82,83]. *P. aeruginosa* T3SS translocates four exoenzymes (ExoS, ExoT, ExoY, and ExoU) into host cells (Figure 2). These exoenzymes have overlapping, yet distinct pathways to target cytoskeleton components and associated junctional complex, causing cell morphological changes and intercellular junction disruption, leading to a loss of barrier integrity. The interactions of these type III exoenzymes with cytoskeleton components are important in the pathogenesis of *P. aeruginosa* infection.

#### 4.1.1. ExoS

ExoS has been studied extensively with several clearly defined eukaryotic targets [84,85,86]. ExoS has a Rho GTPase activating domain (RhoGAP) encoded within the N-terminus and a ADP-ribosyltransferase domain (ADPRT) within the C-terminus [84,87,88]. Through these two domains, ExoS targets cytoskeletal components in different host cell types, including neutrophils, leukocytes, and epithelial cells [89]. The N-terminal Rho GTPases are critical for actin polymerization and cytoskeletal dynamics [90]. The N-terminal domain of ExoS is a mimic of eukaryotic RhoGAP domain so that it can prevent small GTPases Rho, Rac, and Cdc42 from activation by keeping them in inactivate GDP-bound form [31,87,91]. Expression of the N-terminal RhoGAP domain in cultured cells stimulates reorganization of actin stress fibers, contributing to the collapse of the actin cytoskeleton and rounded cellular phenotype [31]. The C-terminus of ExoS encodes an ADP-ribosyltransferase (ADPRT) domain which becomes activated after binding to a eukaryotic cofactor (FAS, factor activating ExoS) [92]. This domain is able to ADP-ribosylate numerous substrates [84]. ADP-ribosylation of Ras and Rab proteins causes a disruption of the actin cytoskeleton, endocytosis, and vesicular trafficking [88,93,94]. In addition, this domain is also responsible for ADP-ribosylation of a set of proteins that link the plasma membrane to the actin cytoskeleton, including ezrin, radixin, and moesin proteins (ERMs), which is implied in the disruption of Rho signaling, resulting in cytoskeletal rearrangements. 

#### 4.1.2. ExoT

Closely related to ExoS, ExoT is also a bifunctional exoenzyme, possessing a RhoGAP domain on its N-terminus and an ADP-ribosylation domain on its C-terminus [95]. The RhoGAP activities of ExoT appear to be biochemically and biologically similar to that of ExoS, targeting substrate such as Rho, Rac, and Cdc42 [29,91,95]. Similar to ExoS, the overexpression of the ExoT RhoGAP domain induces the actin cytoskeleton disruption through a Rho-dependent pathway. On the other hand, while ExoS can ADP-ribosylate a wide range of host proteins, ExoT possesses limited ADP-ribosyltransferase activity. When overexpressed in cultured cells, ExoT ADPRT affects the host cell phagocytic activity while ExoS ADPRT has a cytotoxic effect. One substrate of ExoT ADPRT are Crk adaptor proteins that are essential in signal transduction and are involved in actin reorganization [96,97]. ADP-ribosylation of Crk proteins prevents their interaction with focal adhesion proteins and with DOCK180 which is a guanine nucleotide exchange factors (GEF) for Rac, thus inhibiting Rac-dependent phagocytosis [98]. The ADPRT domain is required for ExoT-induced inhibition of migration and wound healing in epithelial cells [99]. ExoT has been suggested in the regulation of cytoskeletal reorganization as different ExoT mutants differentially affect the subcellular localization of paxillin and focal adhesion kinase [100]. It is noteworthy that, although ExoT ADP-ribosylates a more restricted subset of substrates, it has been suggested that ExoT is important for P. aeruginosa to achieve full virulence in a mouse pneumonia model [30]. A prevalence study of type III secretion genes suggests that nearly all clinical isolated P. aeruginosa strains encode ExoT, while exoS, exoU, and exoY genes were variably expressed [101], suggesting that ExoT may have a more conserved role in the context of P. aeruginosa pathogenesis.

#### 4.1.3. ExoY

ExoY is a nucleotidyl cyclase that synthesizes cyclic nucleotides including cGMP, cAMP, cUMP, and cCMP [102,103,104,105]. ExoY is highly prevalent in clinically isolated strains and has been indicated as an important edema factor which significantly contributes to end-organ dysfunction in critically ill patients with P. aeruginosa lung infection [101,106,107]. Excessively generated cyclic nucleotides can alter cell morphology through disrupting signaling involved in cytoskeletal organization. However, ExoY, to prevent toxicity to bacterial cells, is produced in its inactive form by P. aeruginosa. The mechanisms by which ExoY produces large quantity of cyclic nucleotides in eukaryotic cells are poorly understood. Recent studies reveal ExoY activation upon binding to F-actin in host cells [108]. Binding with F-actin drives ExoY go through a con-formational change which is critical to increase ExoY catalytic activity and generate excessive cyclic. These data indicate that binding to actin filaments (F-actin), but not globular actin (G-actin), activates ExoY, which in turn helps to stabilize actin filaments [108,109,110]. In addition, it has been shown that ExoY promotes Tau phosphorylation which dissociates from microtubule and results in microtubule breakdown, leading to gap formation and increased permeability [103,106]. 

#### 4.1.4. ExoU

ExoU is mutually exclusively expressed with ExoS by *P. aeruginosa* [111,112,113]. ExoU is a phospholipase that induces acute cytotoxic effects and is capable to destroy cell monolayers in a short time period [114]. ExoU production is associated with accelerated lung injury and is often associated with the most severe pathological outcomes in experimental animals and in patients [111,115]. ExoU has been reported to associate with membrane fractions and resides on cell–cell junctions in A549 cells [116]. ExoU presents a high affinity to phosphatidylinositol 4,5-bisphosphate (PI(4,5)P2). PI(4,5)P2 is a multifunctional phosphoinositide located in the eukaryotic plasma membrane and is involved in the regulation of focal adhesion formation and cytoskeletal dynamics. Studies have shown that a high affinity of ExoU with PI(4,5)P2 and subsequent cleavage of PI(4,5)P2 at focal adhesion complexes contribute to the collapse of the cytoskeleton network in human epithelial cells [117]. Although ExoU lipase activity appears to have a large range of substrates in cell cytosol where it may damage plasma membrane components, whether there is physical association between ExoU and junctional proteins or with cytoskeletal components still require further investigation. 

#### 4.1.5. T3SS Needle Tip Complex

The needle tip complex is important in early infection stages as it functions to inject *P. aeruginosa* effectors into host cells upon contact. In addition to its needle-like function, the presence of this needle complex has been reported to induce actin stress fiber formation in cultured murine pulmonary microvascular endothelial cells [118,119].

### 4.2. Regulation of Lung Permeability by P. aeruginosa Secreted Virulence Factors

#### 4.2.1. Elastase

*P. aeruginosa* elastase (PE) is a secreted metalloproteinase with highly efficient proteolytic activity on a number of host structural proteins in airway epithelium [120,121,122,123,124,125]. It has been reported that PE can transiently disintegrate and redistribute tight junction proteins OCLN and ZO-1, induce cleavage of VE-cadherin, and cause actin cytoskeleton reorganization [22,23,62,126,127,128]. By using the B.V strain that is known for its high elastase activity, it has been shown that PE is capable of completely degrading ZO-1 and significantly degrading OCLN [127]. Besides targeting on tight junction proteins, PE has tissue-damaging activities. In addition, PE can degrade lung elastin, an important structural protein for maintaining blood vessel integrity [123,129], as well as matrix proteins including laminin and collagen (type III and type IV), leading to basement membrane impairment [130,131,132]. 

#### 4.2.2. Exotoxin A

*P. aeruginosa* produces a highly toxic virulence factor exotoxin A (ExoA) which is released into extracellular medium by type 2 secretion system (T2SS) [133,134]. It has ADP-ribosylation activity and affects the protein synthesis processes in host cells. ExoA has been shown to delay wound repair in the animal cutaneious injury model through its effects on cytoskeleton remodeling [135]. Treatment with ExoA reduces TJs proteins ZO-1 and ZO-2 and increases paracellular permeability in type II pneumocyte cultures [23]. However, the exact mechanism undergoing ExoA-mediated epithelial barrier damage still need further studies.

### 4.3. Regulation of Lung Permeability by P. aeruginosa Surface-Bound Virulence Factors

#### 4.3.1. Pilus and Flagellum

Type IV pilus and flagellum are important surface structural components for *P. aeruginosa* attachment to cell surface and are critical in preparation for T3SS toxin injection [136,137]. Due to the nature as *P. aeruginosa* surface structure, pilus and flagellum are likely to have roles beyond mediating an initial attachment to the host surface. Evidence show that pilus and flagellum are required for transmigration across epithelial cell junctions [136,137]. Recently, pilus has been shown to preferentially interact with the cell basolateral domain and T3SS effectors are only injected into host cells through their basolateral membrane domain [136,137,138]. Internalization of *P. aeruginosa* in the epithelial basolateral surface requires flagellum binding to heparan sulfate, with subsequent signaling activation of epidermal growth factor receptor (EGFR), phosphoinositide 3-kinases (PI3K), and protein kinase B (AKT) [138]. These findings suggest these surface-bound virulence factors may play an important role in mediating *P. aeruginosa* transmigration through paracellular route. 

#### 4.3.2. Lipopolysccharide

Lipopolysccharide (LPS) is a major structure component which is integrated in the *P. aeruginosa* cell wall and plays an important role in bacterium–host interactions [139]. LPS is a pro-inflammatory mediator which can increase airway epithelial permeability [140]. LPS-induced F-actin rearrangement and actin assembly are important for LPS signaling [59]. However, molecular mechanisms for LPS-induced endothelial cell permeability are still not well understood.

### 4.4. Regulation of Lung Permeability by Quorum Sensing and Other P. aeruginosa Virulence Factors

Quorum sensing (QS) is a specialized cell density-dependent regulation system in bacteria [141,142,143]. These bacterial signals also modulate mammalian airway epithelial cell responses to the pathogen in a process called interkingdom signaling. N-(3-Oxododecanoyl)-L-homoserine lactone (C12) is a small molecule quorum-sensing signal produced by a *P. aeruginosa* lasR-lasI QS system [144,145]. In addition to the regulation of *P. aeruginosa* population behavior, C12 also regulates a range of complex biological processes in host cells. In human epithelial Caco-2 cells, C12 induces a decrease in transepithelial electrical resistance (TER), an increase in paracellular flux, a reduction in the expression and distribution of ZO-1 and OCLN, and reorganization of F-actin through activation of p38 and p42/44 pathways [146]. In intestinal epithelial cells, C12 alters the phosphorylation status of cell junctional components, including E-cadherin, beta-catenin, OCLN, ZO-1, and ZO-3, and JAM-A. In addition, the changes in phosphorylation status of regulatory proteins disrupt the association between junctional components and result in a loss of epithelial barrier and increased paracellular permeability [24,147]. C12 also induces degradation and de-location of TJs proteins (OCLN and tricellulin) in intestinal epithelial Caco-2 cells [148]. These findings collectively indicate that C12 induces epithelial paracellular permeability possibly through a mechanism that mediates the disassembly of intercellular links. C12 induces myofibroblast differentiation in vitro and in vivo for accelerated wound healing [149]. In cultured nonpolarized airway epithelial cells, C12 induces massive morphological changes of cell structure with perturbed gap junction shortly after application [150]. C12 may also facilitate dissemination of virus into bloodstream [151].

#### Rhamnolipids

*P. aeruginosa* produces biosurfactants called rhamnolipids [152,153]. Rhamnolipids act as a potent detergent and have been reported to disrupt intercellular junctions in sheep tracheal epithelium at high concentrations [154]. Rhamnolipids induce ciliostasis of airway epithelial cells and may disrupt their barrier function, allowing invasion of pseudomonas [12]. Alzheimer’s disease (AD) has been attributed to chronic bacterial infections, and the levels of rhamnolipids in sera and cerebrospinal fluid of AD patients are significantly increased when compared to controls [155]. However, the meaning of the increased rhamnolipids levels in AD patients and AD pathogenesis is unclear so far. 

## 5. Conclusions

**Figure 3:**
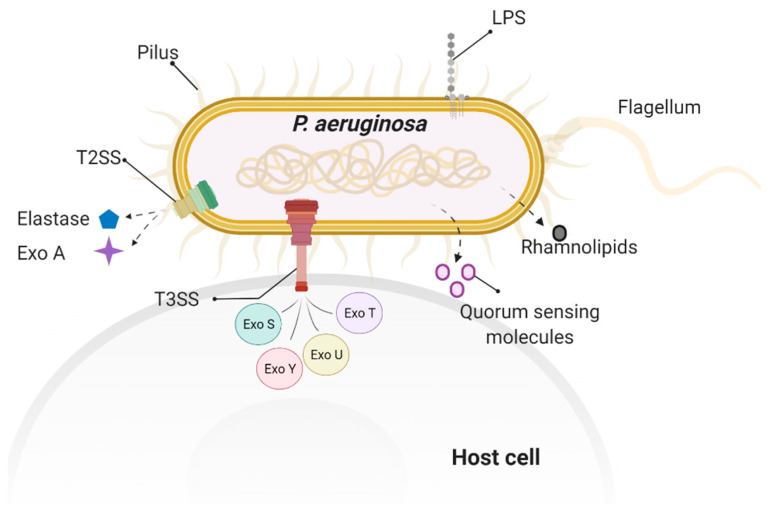
Virulence factors involved in *P. aeruginosa*-induced cytoskeleton rearrangement and impaired barrier integrity. These *P. aeruginosa* virulence factors include surface factors, such as flagellum, pilus, and LPS; secreted factors, such as type III secretion system (T3SS) exoenzymes (ExoS, ExoT, ExoY, ExoU) and rhamnolipid; and quorum-sensing factor, such as N-(3-Oxododecanoyl)-L-homoserine lactone.

**Figure 4:**
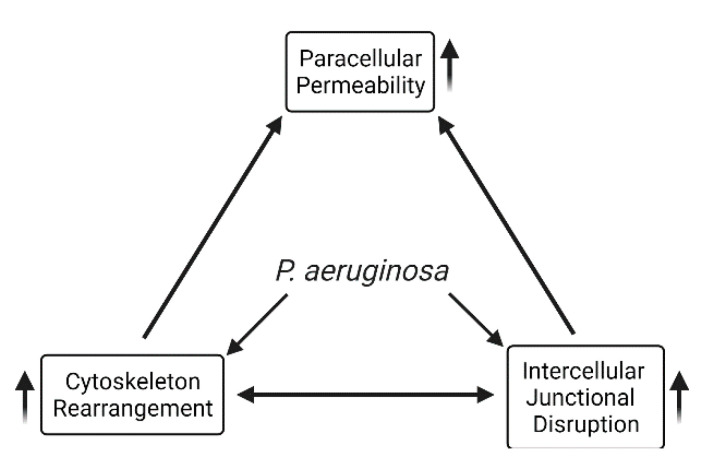
Overview of effects of *P. aeruginosa* virulence factors on intercellular junctional impairment, cytoskeleton rearrangement, and lung barrier dysfunction.

*P. aeruginosa* virulence factors have a significant impact on host biological functions by targeting different cellular components. Figure 3 shows the major virulence factors involved in *P. aeruginosa*-induced cytoskeleton rearrangement and impaired barrier integrity. The actin cytoskeleton plays an important role in coordinating junctional components with cytosolic signaling regulators. Pathological modulation of regulators involved in actin cytoskeleton reorganization links to lung barrier dysfunction. Rho GTPases play critical roles in the regulation of cytoskeleton contractility and dynamics. Virulence factors with the capacity to interfere host Rho GTPases activities can thus disrupt junctional functions due to altered cytoskeleton contractility. For example, ExoS and ExoT can hijack Rho GTPase signaling pathway, by mimicking the eukaryotic Rho GTPases at its N terminal domain, directly altering the function of host cytoskeletal regulation. In addition to delivering cytotoxic type III secretion exoenzymes into eukaryotic cells, other virulence factors, such as surface bound structure LPS, also induce stress fiber formation and cytoskeletal protein reorganization. Furthermore, *P. aeruginosa* produces a large number of exo-products, of which the quorum-sensing molecule, C12 transkingdomly, interferes with host junctional protein expression and redistribution. Hence, as cytoskeleton alteration can be induced at each stage of *P. aeruginosa* infection, a barrier breach can also occur at different levels, through initial bacteria attachment, to toxin injection into cytosol that interferes host cytoskeletal components. Figure 4 shows an overview of effects of *P. aeruginosa* virulence factors on intercellular junctional impairment, cytoskeleton rearrangement, and lung barrier dysfunction. Future studies are needed to elucidate the mechanisms by which virulence factors disturb the mammalian cytoskeleton network and modulate invasion, and to highlight how those activities contribute to the pathogenesis of *P. aeruginosa* infection.

Although *P. aeruginosa* virulence factors use the cytoskeleton network as a common route to modulate barrier integrity and signal transduction, different strategies have been adopted due to distinct features present in epithelial and endothelial cells. The diversified virulence factor types further complicate the pathogenic pathways. Therefore, each virulence factor establishes a unique pathogenic strategy to penetrate lung barrier, and a number of molecular mechanisms have been proposed regarding how *P. aeruginosa* virulent factors breach lung barrier functions. However, appropriate in vivo models are not easily applicable, and most of these mechanisms were based on observations using in vitro cell cultures. One pitfall of in vitro studies is the use of relatively high concentration of cytotoxins, which may not adequately reflect the exact situations in the course of infection. The in vivo condition is confounded with abundant immune cells and fluid flow, which in turn further complicates the interactions between *P. aeruginosa* virulence factors, cytoskeleton, and its associated components. Moreover, the biological effects of virulence factors vary depending upon the route of bacterial delivery and the nature of the host cell types, and different route of bacterial delivery can further affect the biological effects of *P. aeruginosa* virulence factors. 

It is currently not well understood by what mechanisms, and to what degree, TJs or AJs proteins are affected by the destructive effects of *P. aeruginosa* virulence factors. Recent evidence suggests that actin assembly at TJs and AJs are regulated through distinctive mechanisms [71,72]. This is further complicated by the fact that many cytoskeletal regulatory proteins involved in *P. aeruginosa* infection have other roles in cell biology. Future studies will be needed to understand the underlying mechanisms. Furthermore, tight junctions contain at least 40 different proteins [155]. Besides the aforementioned transmembrane proteins (such as CLDNs and OCLN) and adaptor protein (such as ZO-1), other intracellular proteins (such as cingulin, MAGI-1, Pals1, and TATJ) also form scaffolds between transmembrane junctional proteins and the actin cytoskeleton. Whether these proteins are manipulated by *P. aeruginosa* and associated consequences on barrier regulation is unknown. While this review focuses on the role of actin cytoskeleton in regulating lung barrier and permeability, other cytoskeleton structures, such as microtubules, may also play a role in *P. aeruginosa*-induced barrier dysregulation. Additional research and a better understanding of the effects of *P. aeruginosa* virulence factors on lung epithelial and endothelial barrier functions will be important for uncovering novel strategies to reduce *P. aeruginosa*-induced edema and bacteremia. A better understanding of how actin cytoskeleton controls intercellular junction assembly will provide new insights to selectively modulate the paracellular flux between airway epithelial and endothelial cells, which would, in turn, benefit the development of small molecule and proteins for novel therapeutics against *P. aeruginosa*-induced lung complications.

## Data Availability

This is a review and does not include any data.
